# Pervasive TBI and Inhibitory Control in a Male New Zealand Prison Population

**DOI:** 10.3390/brainsci16060637

**Published:** 2026-06-15

**Authors:** Sam Guy, Susan Mahon, James Webb, Makarena Dudley, Alice Theadom

**Affiliations:** 1 Brain Health Research Institute, Faculty of Health and Environmental Sciences, Auckland University of Technology, Auckland 1010, New Zealandjames@webbpsychology.co.nz (J.W.);; 2 School of Psychology, The University of Auckland, Auckland 1010, New Zealand

**Keywords:** traumatic brain injury, executive function, inhibition, prison, cognitive, CWIT, OSU-TBI ID

## Abstract

**Highlights:**

**What are the main findings?**

- A New Zealand male prison population reported high prevalence and incidence of traumatic brain injury (TBI).
- TBI frequency and severity were not associated with poorer cognitive performance.
- Pervasive TBI (clustered/repetitive injury exposure) predicted impaired inhibition.
- Inhibition deficits were identified using the Color-Word Interference Test.

**What are the implications of the main findings?**

- TBI pervasiveness may represent an important factor in the rehabilitative profile of people in prisons.
- Future research is needed to better understand the role of pervasive TBI history on cognitive recovery.

**Abstract:**

**Objective**: Traumatic brain injury (TBI) is disproportionately prevalent in incarcerated populations, yet the potential impact on cognitive functioning remains underexplored. This study examined the relationship between TBI history and cognitive performance in a male prison population. **Method**: Sixty-three participants from Tongariro Prison completed a comprehensive neuropsychological assessment including measures of executive function, memory, processing speed, and perceptual reasoning, with embedded performance validity metrics. TBI history was assessed using the Ohio State University TBI Identification Method (OSU-TBI ID), premorbid function was assessed using the Speed and Capacity of Language Processing (SCOLP) Spot-the-Word task, mood was assessed using the Depression, Anxiety and Stress Scales (DASS-21), and alcohol and substance use were measured using the Alcohol, Smoking and Substance Involvement Screening Test (ASSIST). Regression analyses explored the relationship between TBI history and cognitive functioning, controlling for premorbid function, mood, alcohol and substance use, and ethnicity. **Results**: Contrary to hypotheses, TBI frequency and severity were not associated with poorer cognitive performance in this population. However, a self-reported history of pervasive TBI—defined as repeated head impacts over a narrow time frame—was significantly associated with reduced performance on the Color–Word Interference Test (CWIT) inhibition task, indicating links to greater cognitive disinhibition. **Conclusions**: Findings suggest that experiencing at least one period of pervasive TBI may be associated with an impact on inhibition (but not other aspects of executive functioning) in men in prison. These results underscore the importance of nuanced TBI history assessment and highlight inhibition as a potential target for rehabilitation in incarcerated individuals exposed to repetitive head trauma.

## 1. Introduction

Traumatic brain injury (TBI) is increasingly recognized as a significant public health concern, with increasing evidence of a link to longer term cognitive functioning and increased risk of neurodegenerative diseases [1,2,3,4,5,6]. International research has consistently demonstrated that individuals in prison are disproportionately affected by TBI compared to the general population [7,8,9,10,11]. In the Aotearoa New Zealand context, 64% of male prisoners reported experiencing at least one TBI [9], contrasting the 13% prevalence reported in the general New Zealand population [12]. Moreover, prison populations are more likely to experience repetitive and early-life TBIs, often associated with interpersonal violence, substance use, and socioeconomic disadvantage [13,14,15].

Despite the high prevalence of TBI in correctional settings, the potential cognitive consequences of these injuries remain poorly understood. The existing literature is methodologically heterogeneous, with inconsistent definitions of TBI severity and varied approaches to cognitive assessment [8,16,17]. Studies of offenders have reported conflicting findings, with some identifying associations between TBI and poorer cognitive functioning in domains such as executive functioning [13,18], while others show no significant relationship [19] or even enhanced performance in certain domains [20]. Furthermore, New Zealand prison populations have larger indigenous Māori populations with whom many cognitive assessments, particularly language-reliant assessments, show reduced validity [21,22,23]. These discrepancies highlight the need for more nuanced investigations into the relationship between TBI history and cognitive functioning in prison populations.

This study aimed to address these gaps by examining the association between TBI history and cognitive functioning—including frequency, severity, and pervasiveness—in a sample of male prisoners within Tongariro Prison, a minimum to low–medium security facility in Tūrangi, New Zealand. Compared to the overall NZ prison population, Tongariro Prison is distinct in its security level, the lack of remand prisoners, and in its offense profile. The Tongariro Prison population has more than double the prevalence of sexual assault convictions (50.9%) when compared to the overall NZ prison population (21.0%) (Department of Corrections, Personal Correspondence, 31 January 2024). It was hypothesized that increased exposure to TBI, particularly increased frequency, severity, and exposure to pervasive injuries, would be associated with lower cognitive performance, especially in tasks assessing executive function.

## 2. Materials and Methods

This study employed a cross-sectional design. A one-tailed a priori sample size calculation was conducted for a multiple linear regression model including nine predictor variables. Assuming α=0.05, power of 0.80, and an effect size of $f^{2}=0.12$, the minimum required sample size was estimated to be N=54. A one-tailed power calculation was used because the study was based on directional hypotheses derived from the prior literature, specifically that greater TBI exposure (frequency, severity, and pervasiveness) would be associated with poorer cognitive performance; thus, statistical power was concentrated on detecting effects in the predicted direction.

Participants were recruited through internal prison mail advertisements, with support from the site healthcare team. Eligibility criteria included being aged 18 years or older, English-speaking, able to hold a pen or pencil, and providing informed consent.

Lifetime TBI history was assessed using the OSU-TBI ID, a structured interview tool. The tool captured TBI frequency, severity (based on reported loss of consciousness and post-traumatic amnesia), and pervasiveness. Pervasiveness was identified with Step 3 of the OSU-TBI ID “Have you ever had a period of time in which you experienced multiple, repeated impacts to your head (e.g., history of abuse, contact sports, military duty)?”, with affirmative responses being categorized as positive for a period of pervasive injury [24].

A bespoke neuropsychological assessment was designed to assess multiple cognitive domains while minimizing language dependence and logistical constraints of the prison environment.

- Executive Functioning: Neuropsychological Assessment Battery (NAB) Mazes, NAB Judgment, Delis–Kaplan Executive Function System (D-KEFS) Color–Word Interference Test (CWIT), Color Trails Test.
- Memory: California Verbal Learning Test—Third Edition (CVLT-3).
- Processing Speed: Wechsler Adult Intelligence Scale—Fourth Edition (WAIS-IV) Coding and Symbol Search.
- Perceptual Reasoning: WAIS-IV Matrix Reasoning and Picture Completion.
- Working Memory: WAIS-IV Digit Span (Backwards and Sequencing tasks).

To account for issues of cross-cultural validity, age-stratified norms for Māori were applied to the WAIS-IV items, while others were scored according to their guidelines [25]. Performance validity was assessed using two embedded measures: the Reliable Digit Span—Revised (RDS-R) and the CVLT-3 Forced Choice Recognition trial. Participants were included in the final analysis if they met the validity threshold (RDS-R > 9 or CVLT-3 Forced Choice > 14) on at least one of these measures [26,27]. This performance validity approach was selected to maximize inclusion of valid data while maintaining specificity, given that the study population was expected to experience multiple sociocultural factors, such as differences in education, acculturation, and income that may influence engagement with the assessment battery, and are known to affect cognitive test performance, particularly in minority ethnicity groups [22].

To control for potential confounders related to premorbid functioning, mood, and substance use history, participants completed the Speed and Capacity of Language Processing (SCOLP) Spot-the-Word task, the Depression, Anxiety, and Stress Scale (DASS-21), and the Alcohol, Smoking, and Substance Involvement Screening Test (ASSIST). The SCOLP Spot-the-Word task was used to assess premorbid function in participants. The Spot-the-Word is an assessment of crystallized verbal intelligence, which shows resilience to cognitive decline associated with age or injury [28]. Although Spot-the-Word norms are only provided up to age 65, there is a body of evidence for its validity in older populations [28,29,30], and the New Zealand context specifically [31]. The DASS-21 is a widely used, self-report questionnaire of past-week emotional status, comprising depression, anxiety, and stress domains [32]. The DASS-21 subscales are psychometrically sound, showing high internal consistency and construct validity [33,34,35]. The ASSIST is a screening tool to assess alcohol and substance use history and associated risk [36,37]. The ASSIST captures both lifetime substance use behaviors and past-three-month behaviors across eight items, assessing substance use frequency, cravings, concerns of others, and substance use-associated problems including financial, legal, and social [36,37]. The ASSIST shows good psychometric properties, including high internal consistency, discriminant validity between substances, and high predictive validity for lifelong substance-related difficulties [36,38], with some evidence for validity with New Zealand specific populations [39].

Age and education were controlled through score standardization procedures and were therefore not included in the regression models. Linear regression models were used to determine if group differences by TBI history remained after controlling for ethnicity, premorbid function, mood, and history of alcohol/substance use. Pearson correlation coefficients were used to assess the grouping of cognitive tasks into domains; only processing speed showed sufficient inter-correlation to justify grouping (*r* = 0.63, *p* < 0.01, two-tailed), while all other correlations were non-significant at the 0.05 level or below *r* = 0.60. Accordingly, analyses were conducted for each cognitive task separately, with the exception of WAIS-IV Coding and WAIS-IV Symbol Search, which were combined to form a processing speed outcome. The cognitive performance variables of interest were therefore WAIS-IV Digit Span, CVLT-3 Immediate Recall, CVLT-3 Delayed Recall, D-KEFS Color Trails, NAB Judgment, WAIS-IV Picture Completion, NAB Mazes, WAIS-IV Matrix Reasoning, D-KEFS CWIT, and processing speed (derived from WAIS-IV Coding and Symbol Search).

The Benjamini–Hochberg procedure was used to control the false discovery rate across the multiple tests, while balancing the risk of Type I error and maintaining statistical power in this analysis. Separate Benjamini–Hochberg corrections were applied to analyses by TBI frequency, severity, and pervasiveness, as these variables represent conceptually distinct dimensions of injury exposure corresponding to independent families of hypotheses with cognitive outcomes.

## 3. Results

Of the 71 men who attended an interview, 63 completed (or partially completed) the assessment and met performance validity criteria. Two participants did not complete the assessments due to language difficulties. Data for six participants were excluded due to not meeting performance validity criteria. Study sample representativeness was assessed through chi-square comparisons with the overall Tongariro Prison population. The final sample broadly reflected the age and ethnicity distribution of the Tongariro Prison population, with a mean age of 48.3 years (SD = 14.14) and 52.4% identifying as Māori and 46.0% as European; refer to Table 1 for demographic comparison.

### 3.1. TBI History

Participants reported a high number of lifetime TBIs. Of the participants, 95% reported at least one lifetime TBI. Of the participants, 76.1% reported multiple lifetime TBIs (M = 2.83). Of the participants, 25.4% reported at least one TBI in the moderate or severe range, 49.2% reported a period of pervasive TBI and 30.2% of participants reported multiple periods of pervasive TBI exposure.

### 3.2. Regression Analyses

Multiple linear regression models were used to assess the predictive value of TBI history on cognitive functioning, while controlling for ethnicity, mood, substance use, and premorbid functioning. The observed differences between participants who experienced at least one period of pervasive TBI and those who did not on the CWIT (inhibition) task remained after controlling these factors (Table 2) (β = −0.41, *p* < 0.01, *n* = 57), suggesting those with pervasive TBI history performed lower on this task. Pervasive TBI history remained a statistically significant predictor of task performance after applying the Benjamini–Hochberg procedure to correct for multiple comparisons (adjusted *p* = 0.04). Ethnicity was also a significant of CWIT (inhibition) performance (β = −0.29, *p* = 0.04).

Digit Span (Working Memory): TBI frequency was positively associated with improved performance (β = 0.30, *p* = 0.02), which was no longer a significant association after applying the Benjamini–Hochberg procedure (adjusted *p* = 0.19).

CVLT-3 (Immediate Recall): Increased TBI severity was positively associated with improved performance (β = 0.26, *p* < 0.05), which was no longer a significant association after applying the Benjamini–Hochberg procedure (adjusted *p* = 0.46). TBI frequency was positively associated with improved performance (β = 0.28, *p* = 0.04), which was no longer a significant association after applying the Benjamini–Hochberg procedure (adjusted *p* = 0.19).

No significant associations were found between TBI history and performance on measures of processing speed, delayed memory, perceptual reasoning, or judgment. The Benjamini–Hochberg procedure was applied separately to the TBI severity, frequency, and pervasiveness analyses, each comprising 10 cognitive outcome variables. Raw and adjusted *p*-values are reported in the Supplementary Materials Tables S1–S3.

## 4. Discussion

This study explored the relationship between TBI history and cognitive performance in a New Zealand male prison population. The hypotheses were partially supported. Participants who had been exposed to pervasive TBI had reduced performance on the inhibition trial of the CWIT compared to those who had not. The hypotheses that more frequent and severe TBI would be associated with poorer cognitive functioning were not supported.

Ethnicity also emerged as a significant predictor of CWIT inhibition performance. However, as ethnicity was included as a covariate rather than a primary variable of interest and was not subject to correction for multiple comparisons, this finding should be interpreted cautiously. It may reflect broader sociocultural, educational, or assessment-related influences on test performance, rather than a direct relationship with TBI-related cognitive outcomes.

The impact of repetitive or pervasive TBI history on cognition aligns with prior research in general (non-offending) populations [40,41,42] and animal models [43,44,45]. Inhibition deficits are prevalent in offending populations [46,47,48,49] and the present findings suggest pervasive TBI may show a meaningful association with this. Mechanistically, repetitive TBI is associated with microstructural white matter damage in the prefrontal cortex [50,51,52]. Evidence also indicates a dose–response relationship, with more frequent or clustered injuries predicting greater long-term cognitive issues [42,53]. While the dose–response effect of TBI severity on cognitive sequelae is well established [4,54,55], these results extend the framework by implicating injury pervasiveness as an additional determinant. This finding provides a useful data point that may direct future research in understanding the potential relationship between repetitive, pervasive TBI exposure and anti-social behaviors that are associated with disinhibition. However, it cannot further elucidate the causal direction of this relationship; although some evidence has demonstrated that TBI precedes risk-taking behavior [56], this requires further investigation. It is also important to note that the relationship between cognitive disinhibition, and social disinhibition or anti-social behavior is not entirely clear, with a body of evidence showing that these are distinct and inconsistently correlated phenomena [57,58,59,60,61,62].

The lack of associations between cognitive test performance and TBI frequency may be influenced by difficulties that high TBI incidence populations experience in remembering exactly the number of injuries that they have sustained over their lifetime. This suggests a potential limitation of self-reported TBI frequency as an index of history in high-incidence populations. The lack of significant associations between TBI severity and cognitive performance is also notable. This finding contrasts with studies in general populations where moderate-to-severe TBI is consistently linked to long-term cognitive deficits [55,63,64,65,66,67]. The discrepancy may be due to the unique characteristics of prison populations, including resilience, comorbidities, or methodological differences in TBI reporting and classification.

The finding that pervasive TBI, rather than frequency or severity, was associated with cognitive impairment supports calls for more nuanced assessments of TBI history [40,68]. Pervasive TBI, often resulting from repeated head impacts in contexts such as domestic violence, fighting, or contact sports, may have cumulative effects. It was observed in this study that many participants experience multiple periods of pervasive TBI over their lifetime which suggests that the OSU-TBI ID could be refined to reflect this difference in participants histories, particularly when the tool is being used with high-incidence populations such as those in prison, domestic violence survivors, and the housing-insecure.

This study found that inhibition was particularly affected by pervasive TBI. Inhibition deficits have been linked to violent and sexual offending [69,70,71]. The CWIT’s sensitivity to these impairments suggests it may be a valuable tool in forensic neuropsychological assessment for men in prison. This supports previous research which has demonstrated CWIT sensitivity to executive functioning impairments in forensic and psychiatric populations and these findings provide further support for the utility of this tool with vulnerable populations [47,72,73,74].

### 4.1. Limitations

Several limitations of this study must be acknowledged. The reliance on self-reported TBI history introduces potential recall bias, particularly in a population with high rates of early-life and repetitive injuries [75,76]. The cross-sectional design limits causal inference and the results cannot be generalized to females in prison. Although the study sample was demographically representative of the Tongariro Prison population, the unique demographic profile of Tongariro Prison—older age and with higher rates of sexual offending—limits generalizability. The offense profile of the study sample was also not recorded, and we therefore cannot be certain whether the offense types of the study sample aligned with the overall Tongariro Prison population. The modest sample size relative to the number of predictors also introduces potential risk of model overfitting and reduced stability of estimates, and findings should be interpreted with caution. While the cognitive assessment battery was designed to optimize validity with a New Zealand and disproportionately New Zealand Māori study population, it must also be acknowledged that cross-cultural validity remains somewhat tenuous—particularly for assessments of memory, such as the CVLT-3 [77,78]. Finally, pervasive TBI in this study was derived from a single OSU-TBI ID item and does not fully capture the frequency, severity, cause, or temporal patterning of injuries; a more comprehensive construct of pervasiveness would ideally integrate and weight these factors.

### 4.2. Implications and Future Directions

These findings highlight the potential importance of determining exposure to pervasive TBI when assessing TBI history in men in prison. The findings also highlight that previous studies which have not identified any impacts of TBI history on cognition in prison may be limited if inhibition was not included as an outcome. Future research should aim to replicate these results in larger and more diverse samples, including women and younger offenders [79,80]. Longitudinal studies would help clarify the directionality of the relationship between TBI and cognition, to help inform targeted rehabilitation strategies.

## 5. Conclusions

This study investigated the relationship between TBI history and cognitive functioning in a New Zealand male prison population. While no significant associations were found between cognitive performance and TBI frequency or severity, a history of pervasive TBI was significantly associated with reduced performance on the CWIT (inhibition). This finding suggests that pervasive TBI may represent a marker of vulnerability in inhibitory control; however, given the cross-sectional design and the specificity of the effect, further research is needed. These results highlight the importance of further research that may better characterize the impact of repetitive head trauma, particularly through larger and longitudinal studies, and determine whether targeted assessment or intervention approaches may be worthwhile.

## Figures and Tables

**Table 1:** Study sample demographics.

		Study Sample *N* = 63 (%)	Total Tongariro Prison Population Jan 2024 *N* = 379 (%)
Age in years	<30	5 (7.9)	48 (12.7)
	30–59	45 (71.4)	251 (66.2)
	>59	13 (20.6)	80 (21.1)
Ethnicity	Māori and Indigenous Pacific Islander	34 (54.0)	203 (53.6)
	European	29 (46.0)	154 (40.6)
	Other	0 (0.0)	22 (5.8)

**Table 2:** Multiple linear regression of CWIT inhibition by TBI history.

	Unstandardized B (95% C.I.)	SE Β	Standardized β	*p*
Constant	13.00 (8.83, 17.16)	2.07		<0.01
Number of TBIs	−0.06 (−0.48, 0.37)	0.21	−0.04	0.79
Most Severe TBI	−0.12 (−1.16, 0.91)	0.52	−0.04	0.81
Any Period of TBI Pervasiveness	−2.31 (−3.84, −0.78)	0.76	−0.41	<0.01
Ethnicity	−1.49 (−2.91, −0.07)	0.71	−0.29	0.04
ASSIST	−0.12 (−0.33, 0.08)	0.10	−0.26	0.23
DASS Depression	0.20 (−0.06, 0.47)	0.13	0.35	0.12
DASS Anxiety	0.03 (−0.22, 0.28)	0.12	0.06	0.82
DASS Stress	0.15 (−0.08, 0.37)	0.11	0.18	0.20
SCOLP Spot-the-Word	0.01 (−0.26, 0.28)	0.14	0.01	0.96

## Data Availability

The datasets presented in this article are not readily available due to the privacy and ethics restrictions of working with an identified vulnerable study population. Requests to access the datasets should be directed to S.G.

## Supplementary Materials

The following supporting information can be downloaded at: https://www.mdpi.com/article/10.3390/brainsci16060637/s1, Table S1. Benjamini-Hochberg Adjustment for Multiple Testing of Analysis by TBI Severity. Table S2. Benjamini-Hochberg Adjustment for Multiple Testing of Analysis by TBI Frequency. Table S3. Benjamini-Hochberg Adjustment for Multiple Testing of Analysis by TBI Pervasiveness.

## Abbreviations

The following abbreviations are used in this manuscript:

TBI	Traumatic Brain Injury
OSU-TBI ID	Ohio State University Traumatic Brain Injury Identification
SCOLP	Speed and Capacity of Language Processing
DASS-21	Depression, Anxiety, and Stress Scales (21-item version)
ASSIST	Alcohol, Smoking, and Substance Involvement Screening Test
CWIT	Color Word Interference Test
NAB	Neuropsychological Assessment Battery
D-KEFS	Delis–Kaplan Executive Function System
CVLT-3	California Verbal Learning Test—Third Edition
WAIS-IV	Wechsler Adult Intelligence Scale—Fourth Edition
RDS-R	Reliable Digit Span—Revised
