# Supplementary material for: Pervasive TBI and Inhibitory Control in a Male New Zealand Prison Population
Source: Brain Sci. 2026 Jun 15;16(6):637. doi: 10.3390/brainsci16060637 (PMC13297191; doi:10.3390/brainsci16060637)
Supplement: Supplementary file 1 [file brainsci-16-00637-s001.zip › brainsci-4346944-supplementary.pdf]

Supplementary Material

Benjamini-Hochberg Procedure for Multiple Testing

Table S1. Benjamini-Hochberg Adjustment for Multiple Testing of Analysis by TBI Severity

Cognitive Domain	Unadjusted *p*-value	Adjusted *p*-value
Immediate Recall	0.05	0.43
Mazes	0.10	0.43
Delayed Recall	0.13	0.43
Processing Speed	0.17	0.43
Digit Span	0.22	0.43
Matrix Reasoning	0.42	0.69
Color Trails	0.58	0.82
Picture Completion	0.74	0.82
CWIT	0.81	0.82
Judgement	0.82	0.82

Table S2. Benjamini-Hochberg Adjustment for Multiple Testing of Analysis by TBI Frequency

Cognitive Domain	Unadjusted *p*-value	Adjusted *p*-value
Digit Span	0.02	0.19
Immediate Recall	0.04	0.19
Color Trails	0.14	0.48
Judgement	0.20	0.51
Picture Completion	0.30	0.61
Delayed Recall	0.39	0.65
Processing Speed	0.62	0.79
Mazes	0.77	0.79
Matrix Reasoning	0.77	0.79
CWIT	0.79	0.79

Table S3. Benjamini-Hochberg Adjustment for Multiple Testing of Analysis by TBI Pervasiveness

Cognitive Domain	Unadjusted *p*-value	Adjusted *p*-value
CWIT	0.00	0.04
Matrix Reasoning	0.09	0.44
Immediate Recall	0.32	0.84
Delayed Recall	0.34	0.84
Judgement	0.50	0.84
Digit Span	0.58	0.84
Picture Completion	0.59	0.84
Processing Speed	0.74	0.85
Mazes	0.77	0.85
Color Trails	0.85	0.85
